# Validation of a Manual Method for Measuring Left Atrial Reservoir Strain Against Automated Speckle Tracking Analysis

**DOI:** 10.3390/diagnostics15162073

**Published:** 2025-08-19

**Authors:** Marina Leitman, Vladimir Tyomkin

**Affiliations:** 1 Department of Cardiology, Shamir Medical Center, Zerifin 70300, Israel; 2 Gray Faculty of Medical & Health Sciences, Tel Aviv University, Tel Aviv 69978, Israel

**Keywords:** left atrial strain, reservoir strain, speckle tracking echocardiography, manual measurement, atrial fibrillation, sinus rhythm, strain imaging, left atrial function

## Abstract

**Background:** Left atrial strain, particularly reservoir strain, has emerged as a sensitive marker of left atrial function and an early indicator of diastolic dysfunction and cardiovascular risk. However, automated left atrial strain analysis is not universally available, particularly in resource-limited settings. In this study, we propose a manual method for calculating biplane left atrial reservoir strain and validate its agreement with automated software in patients with atrial fibrillation and in sinus rhythm. **Methods:** Echocardiography examinations from 30 patients with atrial fibrillation and 30 patients in sinus rhythm were analyzed. Left atrial reservoir strain was calculated using both an automatic speckle tracking imaging-based algorithm and a manual point-by-point method based on atrial wall delineation. Agreement between methods was assessed using Pearson correlation, Bland–Altman analysis, and intraclass correlation coefficient. **Results:** Strong correlation and excellent agreement were observed between the two methods in both groups. Pearson correlation coefficients were r = 0.95 (*p* < 0.0001) in the atrial fibrillation group and r = 0.94 (*p* < 0.0001) in the sinus rhythm group. Bland–Altman analysis showed narrow limits of agreement, particularly in the atrial fibrillation group. The intraclass correlation coefficient was 0.95 in atrial fibrillation and 0.92 in sinus rhythm, indicating excellent reliability. The standard error of measurement and minimal detectable change were low in both groups. **Conclusions:** Manual measurement of left atrial reservoir strain is feasible, reproducible, and demonstrates excellent agreement with automated software. It may serve as a reliable alternative in clinical scenarios where automated tools are unavailable.

## 1. Introduction

The left atrium plays a vital role in cardiac function by contributing to left ventricular filling through its reservoir, conduit, and booster pump phases. Among these, left atrial reservoir function—reflecting atrial compliance and myocardial deformation during left ventricular systole—has emerged as a particularly sensitive and early indicator of diastolic dysfunction and atrial remodeling [1,2,3]. The importance of accurate left atrial strain measurement has further increased in light of recent recommendations for diastolic function assessment, in which left atrial strain is incorporated as a key parameter in the diagnostic algorithm [4].

Speckle tracking echocardiography enables quantification of left atrial strain, providing valuable insights into atrial myocardial mechanics beyond those offered by traditional volumetric parameters such as the left atrial volume index. Left atrial strain is less dependent on loading conditions and has demonstrated prognostic value across a spectrum of cardiovascular conditions, including heart failure with preserved ejection fraction, atrial fibrillation, and valvular heart disease [2,5]. Notably, reduced left atrial strain correlates with elevated left ventricular filling pressures and predicts adverse cardiovascular outcomes, even in patients with normal atrial size [3]. In patients with atrial fibrillation, left atrial strain is frequently diminished and has been shown to predict rhythm outcomes following interventions such as cardioversion or catheter ablation [6].

Despite growing recognition of its clinical utility, routine implementation of left atrial strain assessment remains limited. A major barrier is the dependence on proprietary, vendor-specific software for automated or semi-automated atrial tracking. Such tools may not be available in all echocardiography laboratories, particularly in resource-limited settings or when analyzing archived images retrospectively. Furthermore, differences in tracking algorithms across vendors may impair reproducibility and hinder cross-platform comparisons [7].

To address these limitations, a simple, accessible, and reproducible method for measuring left atrial reservoir strain is needed—one that does not rely on advanced software. A manual approach based on visual tracking of the atrial wall may provide a viable alternative, especially in environments where automated tools are unavailable or impractical.

In this study, we introduce and validate a novel manual method for calculating biplane left atrial reservoir strain. We compared manual measurements with automated strain values in patients with sinus rhythm and atrial fibrillation—two cohorts with distinct atrial mechanical profiles. Our goal was to determine whether the manual approach could serve as a reliable substitute for automated strain quantification, thereby expanding the accessibility of this clinically valuable parameter.

## 2. Methods and Materials

### 2.1. Study Population

A total of 60 patients were included in this study: 30 with atrial fibrillation and 30 with sinus rhythm. All patients underwent transthoracic echocardiographic examinations during April and May 2025 at our institution.

#### Inclusion and Exclusion Criteria

Patients were eligible for inclusion if they had undergone a transthoracic echocardiographic examination at our center between April and May 2025 and were found to be in either documented atrial fibrillation at the time of the exam or in stable sinus rhythm. To ensure reliable strain analysis, only studies with adequate image quality—specifically, clear apical four-chamber and two-chamber views of the left atrium without foreshortening or significant artifacts—were included.

Exclusion criteria were as follows: (1) inadequate acoustic window or poor image quality preventing accurate strain measurement; (2) history of cardiac surgery; (3) presence of congenital heart disease; and (4) left atrial thrombus or other structural abnormalities that could interfere with LA strain assessment.

### 2.2. Study Design

All transthoracic echocardiographic examinations were performed by certified echocardiography technicians. All technicians involved in this study held formal certification and had more than 5 years of clinical experience in cardiac ultrasound. All examinations were reviewed by a senior cardiologist with over 10 years of experience in echocardiography for clinical purposes.

Echocardiographic studies were retrospectively retrieved and analyzed offline. Biplane left atrial reservoir strain was measured using both a manual method and a conventional speckle tracking-based automated algorithm. Manual measurements were independently performed by one of the study investigators [M.L.], blinded to the automated strain analysis results. To minimize the risk of measurement error, each manual strain measurement was performed twice per patient, and the average value was used for statistical analysis. Intra-observer and inter-observer variability were assessed and reported using the intraclass correlation coefficient (ICC).

### 2.3. Echocardiography Evaluation

All echocardiographic examinations were performed in accordance with current clinical guidelines [8]. Imaging was conducted using the VIVID E95 ultrasound system (version 203; GE Healthcare), with all studies digitally stored for offline analysis. Standard assessments included linear and volumetric measurements of the cardiac chambers, in line with recommended protocols.

The biplane left atrial volume index was calculated using the following formula

(1) $$\frac{8}{3}\pi *\frac{\left(A1\right)*\left(A2\right)}{L}$$

where A1 and A2 represent the left atrial areas in the apical four-chamber and two-chamber views, respectively, L is the shorter of the two lengths measured in each view. The resulting volume was then normalized to body surface area to obtain left atrial volume index (LAVi).

The left ventricular mass (LVM) was calculated using the following formula

(2) $$0.8*1.4*[{\left(IVS+PW+LVID\right)}^{3}-{LVID}^{3}]+0.6g$$

where IVS is the interventricular septal thickness at end-diastole, PW is the posterior wall thickness at end-diastole, and LVID is the left ventricular internal diameter at end-diastole. The resulting left ventricular mass was then indexed to body surface area to obtain left ventricular mass index (LVMi). Relative wall thickness, RWT, was calculated as

(3) $$\mathrm{RWT}=\frac{2PW}{LVEDD}$$

where PW is the posterior wall thickness, and LVEDD is the left ventricle end-diastolic diameter.

Additionally, left atrial strain was assessed in accordance with the recent consensus document of the European Association of Cardiovascular Imaging [7].

**Figure 1:**
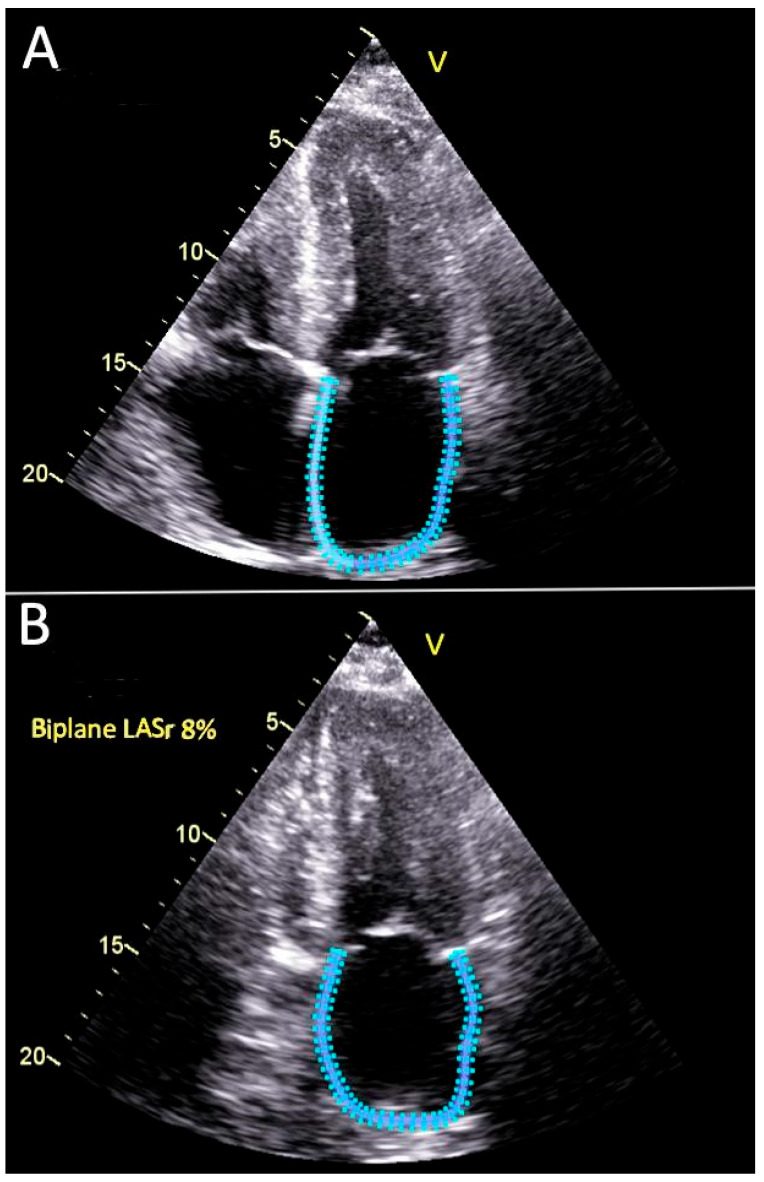
Automated measurement of biplane average peak left atrial reservoir strain. (**A**) Apical 4-chamber view. (**B**) Apical 2-chamber view. The left atrial endocardial border is tracked using an automated speckle tracking algorithm. The calculated biplane left atrial reservoir strain value is shown in panel B, LASr = 8%.

**Automatic Biplane Method for Left Atrial Strain** [7,9], **Figure 1**.

The 4-chamber and 2-chamber apical views were used by the automated software. Optimal, non-foreshortened apical views were utilized, with the reference time point set at left ventricular end-diastole (strain = 0), as determined by the timing of mitral valve inflow. The endocardial border of the left atrial wall was carefully traced. Reservoir strain (LASr) was defined as the peak atrial strain value occurring during left ventricular systole, just prior to mitral valve opening. Average peak reservoir strain was calculated and reported, Figure 1. Both automatic algorithm and manual method were applied.

**Figure 2:**
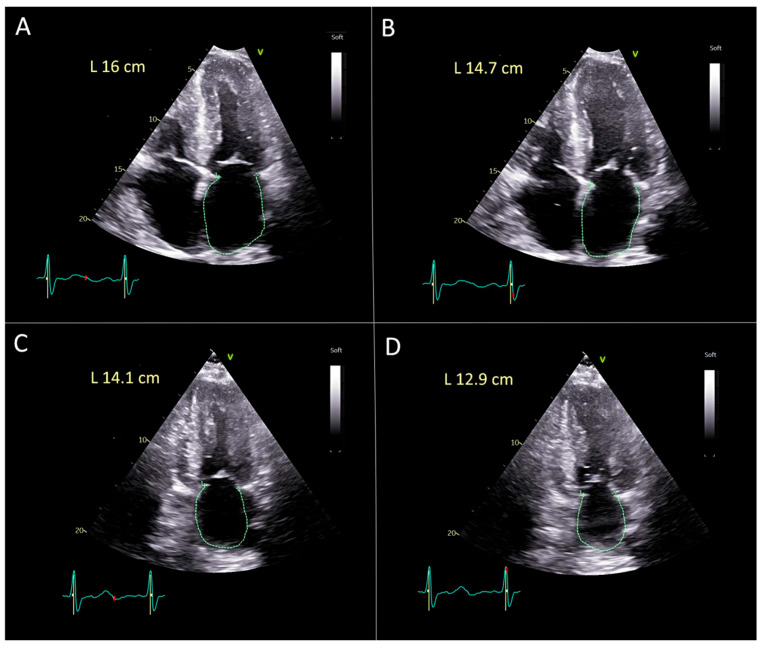
Manual measurements of biplane average peak reservoir left atrial strain. (**A**) Apical 4-chamber view during systole: maximal left atrial length (Ls4c) = 16.0 cm. (**B**) Apical 4-chamber view during diastole: minimal left atrial length (Ld4c) =14.7 cm. (**C**) Apical 2-chamber view during systole: maximal left atrial length (Ls2c) =14.1 cm. (**D**) Apical 2-chamber view during diastole: minimal left atrial length (Ld2c) = 12.9 cm. Manual biplane left atrial reservoir strain (LASr) is calculated using the following formula: Biplane LASr (%) = [((Ls4c − Ld4c)/Ls4c) + ((Ls2c − Ld2c)/Ls2c)]2 × 100. Substituting the values = [((16.0 − 14.7)/16.0) + ((14.1 − 12.9)/14.1)]2 × 100 = [(1.3/16.0) + (1.2/14.1)]2 × 100 ≈ (0.08125 + 0.08511)/2 × 100 ≈ 0.08318 × 100 ≈ 8.3%. This value represents the manually measured biplane LASr.

**Manual Biplane Method for Left Atrial Strain**, **Figure 2**.

The same apical four-chamber and two-chamber views were used for the manual measurements.

Strain was calculated using the following formula:

strain = (L(t) − L(0))/L(0), where L(0) is the initial length and L(t) is the new length at a specific time. Similarly, left atrial peak reservoir strain can be calculated as

(systolic left atrial length − diastolic left atrial length)/systolic left atrial length × 100.(4)

For the calculation of average peak reservoir biplane left atrial strain we used formula

(5) $$\mathrm{Biplane}\;\mathrm{strain}=\frac{\left(\frac{Ls4c-Ld4c}{Ls4c}\right)+\left(\frac{Ls2c-Ld2c}{Ls2c}\right)}{2}$$

where Ls4c is the maximal length of the left atrium during the end systole just before opening of the mitral valve from apical 4-chamber view; Ld4c is the smallest length of the left atrium during the end diastole from the apical 4-chamber view; Ls2c—is a maximal length of the left atrial during the end systole just before opening of the mitral valve at 2-chamber view; Ld2c—is the shortest length of the left atrium during end diastole from 2-chamber view, Figure 2.

### 2.4. Statistical Methods

Descriptive statistics were calculated to summarize the characteristics of each parameter. Continuous variables are presented as mean ± standard deviation, and categorical variables as absolute numbers and percentages. The normality of distribution for continuous variables was assessed using the Kolmogorov–Smirnov test. Comparisons between paired continuous variables were performed using a two-tailed paired *t*-test.

For categorical variables, univariate comparisons were conducted using the Chi-square test or Fisher’s exact test, as appropriate. A *p*-value of <0.05 was considered statistically significant.

Agreement between manual and automated strain measurements was assessed using Pearson correlation coefficients, intraclass correlation coefficients (ICC), coefficient of variation (CV), standard error of measurement (SEM), minimal detectable change (MDC), and Bland–Altman analysis.

All statistical analyses were performed using IBM SPSS Statistics for Windows, Version 28.0 (Armonk, NY, USA: IBM Corp.).

A post hoc power analysis was performed using G*Power version 3.1.9.7 (Heinrich-Heine-Universität Düsseldorf, Germany) to evaluate the adequacy of the sample size for detecting a significant correlation between manual and automated left atrial strain measurements. Assuming a two-tailed test, an alpha level of 0.05, and a large effect size (r = 0.80), the analysis showed that a sample size of 30 patients per group provides greater than 99% power (1-β > 0.99) to detect a statistically significant correlation.

### 2.5. Ethical Approval

The study was approved by the Helsinki Ethics Committee of Shamir (Asaf Harofeh) Medical Center (approval number: ASF-0122-15(V2), dated 22 May 2025). All echocardiographic images included in the analysis were fully de-identified to ensure patient confidentiality. All procedures were conducted in accordance with institutional guidelines and applicable regulations. Due to the retrospective and fully anonymized nature of the study, the requirement for informed consent was waived by the Local Helsinki Committee at Shamir (Asaf Harofeh) Medical Center.

## 3. Results

The demographic and clinical characteristics of all patients are presented in Table 1. The mean age of patients with atrial fibrillation was 79 ± 8.7 years, compared to 47 ± 30.9 years in patients with sinus rhythm. Each group included 19 males (63.3%). Patients with atrial fibrillation had a significantly higher body mass index than those in sinus rhythm (28.3 ± 5.0 vs. 25.7 ± 3.7; *p* < 0.03). Patients with atrial fibrillation had a significantly higher prevalence of heart failure (*p* < 10^−10^), with an average New York Heart Association (NYHA) functional class of II–III, compared to class I in patients with sinus rhythm. Additionally, patients with atrial fibrillation presented with a significantly greater burden of comorbidities, including hypertension, diabetes mellitus, ischemic heart disease, chronic kidney disease, and chronic obstructive pulmonary disease.

Echocardiographic findings for all patients are summarized in Table 2. Patients with atrial fibrillation had significantly lower left ventricular ejection fraction compared to those in sinus rhythm 47.8 ± 10.5% vs. 60.5 ± 1.5%, *p* < 10^−7^. Left ventricular mass index was also significantly higher in the atrial fibrillation group 109.3 ± 35.7 g/m^2^ vs. 78.7 ± 21.2 g/m^2^, *p* < 10^−3^, along with greater relative wall thickness indicating concentric remodeling 0.48 ± 0.14 vs. 0.40 ± 0.10, *p* < 0.01.

Left atrial size was markedly larger in patients with atrial fibrillation compared to those in sinus rhythm, *p* < 10^−7^. Moderate or greater mitral regurgitation was present in over half of the atrial fibrillation group, and tricuspid regurgitation was observed in approximately two-thirds of these patients. Pulmonary artery systolic pressure was significantly elevated in the atrial fibrillation group 44.2 ± 11.0 mmHg vs. 25.2 ± 6.5 mmHg, *p* < 10^−10^.

Left atrial reservoir strain was markedly reduced in atrial fibrillation compared to sinus rhythm. For automatic measurements, left atrial reservoir strain was 6.4 ± 2.3% versus 30.3 ± 4.7%, *p* < 10^−31^, and for manual measurements, 6.5 ± 2.2% versus 29.5 ± 4.4%, *p* < 10^−32^.

### Validation of Manual Method for the Left Atrial Strain Calculation

Pearson correlation was performed separately for each group of patients. In the atrial fibrillation group (*n* = 30), Pearson correlation between manual and automatic biplane left atrial reservoir strain was r = 0.95, *p* < 0.0001, Table 3. In the sinus rhythm group (*n* = 30), the correlation was similarly high r = 0.94, *p* < 0.0001.

**Figure 3:**
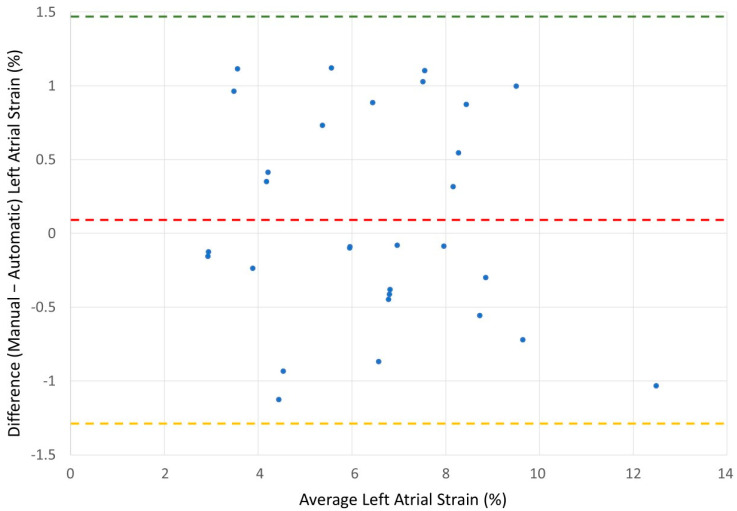
Bland–Altman plot for patients with atrial fibrillation. Bland–Altman plot demonstrating agreement between manual and automatic measurements of biplane left atrial reservoir strain in 30 patients with atrial fibrillation. The mean difference (bias) was 0.094%, with limits of agreement ranging from −1.33% to +1.52%. The horizontal red dashed line represents the bias; the green and yellow dashed lines represent the upper and lower limits of agreement, respectively.

**Figure 4:**
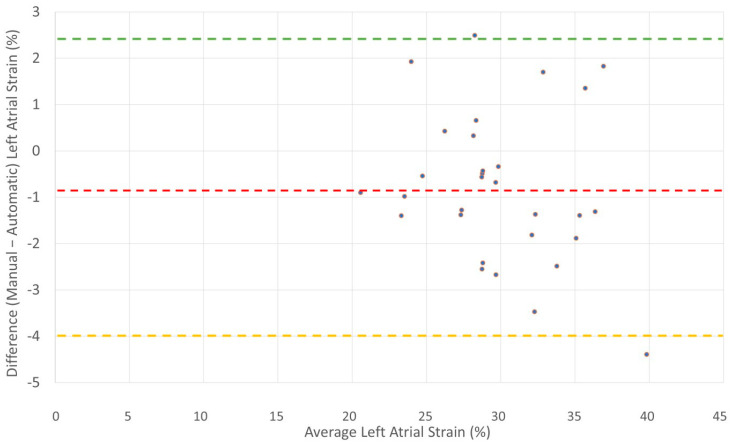
Bland–Altman plot for patients in sinus rhythm. Bland–Altman plot illustrating agreement between manual and automatic measurements of biplane left atrial reservoir strain in 30 patients with sinus rhythm. The mean difference (bias) was −0.8%, with limits of agreement from −4.08% to +2.48%. The horizontal red dashed line represents the bias; the green and yellow dashed lines represent the upper and lower limits of agreement.

We performed a Bland and Altman analysis to estimate the agreement between both methods, Figure 3 and Figure 4. The results show good agreement between the methods in both groups. The atrial fibrillation group has tighter agreement, likely due to the lower strain values and possibly less dynamic left atrial function. The sinus group shows more variability, but this may be due to higher strain values and greater left atrial motion during sinus rhythm.

Intraclass correlation coefficient (ICC2,1) was calculated. For the group of patients with atrial fibrillation it was 0.95, that means excellent agreement. For the group of patients with sinus rhythm it was 0.92, which is also an excellent agreement.

Coefficient of variation for manual-automatic differences was calculated to be 11% for the group of patients with atrial fibrillation and 5.5% for the group of patients with sinus rhythm. The atrial fibrillation group has higher relative variability between manual and automatic values, likely due to the lower absolute strain values. The group of patients with sinus rhythm despite larger absolute differences shows lower coefficient of variation, indicating more consistent agreement relative to the strain magnitude.

Standard error of measurement was 0.16% for patients with atrial fibrillation and 0.46% for patients with sinus rhythm. Minimal detectable change_95_ was 0.43% for patients with atrial fibrillation and 1.27% for patients with sinus rhythm. The standard error and minimal detectable change_95_ indicate high measurement precision in both groups.

The repeatability coefficient was calculated in a predefined 15-patient reproducibility subset. In the sinus rhythm group (*n* = 15), the within-method Bland–Altman repeatability coefficient was 10.53% (95% CI 7.71–16.61%) for the manual method and 5.66% (95% CI 4.15–8.93%) for the automated method. In the atrial fibrillation group (*n* = 15), the repeatability coefficient was 0.82% (95% CI 0.60–1.29%) for the manual method and 0.70% (95% CI 0.51–1.10%) for the automated method.

The correlation and agreement between manual and automatic methods for the calculation of left atrial strain, as well as the repeatability coefficients, are summarized in Table 4.

To better estimate generalizability, we performed Pearson correlation across the entire study population. The correlation coefficient r was 0.995 with a *p*-value < 10^−59^, which indicates an extremely positive correlation. The *p*-value is far below 0.0001, indicating the results are highly statistically significant, Table 5.

**Figure 5:**
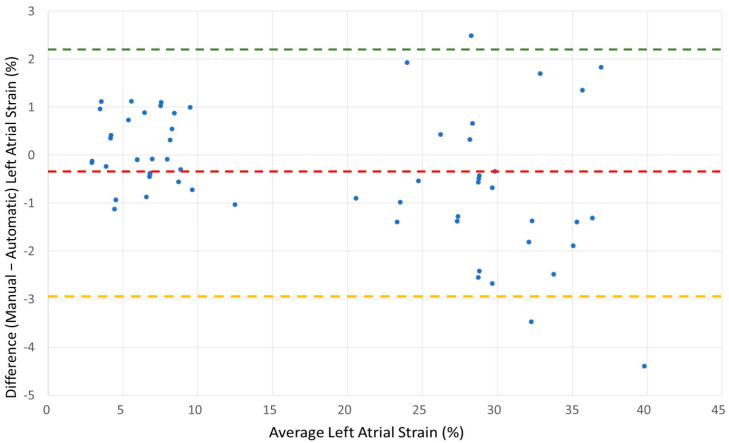
Bland–Altman plot comparing manual and automatic biplane left atrial reservoir strain measurements in all 60 patients (combined atrial fibrillation and sinus rhythm). The mean difference (red dashed line) was −0.35%, with 95% limits of agreement (green and yellow dashed lines) between −2.96% and +2.26%. The x-axis shows the average of manual and automatic measurements, and the y-axis shows the difference between the two methods.

The Bland–Altman analysis for the total cohort was performed, (*n* = 60), Figure 5, and demonstrated a small mean difference of −0.35%, indicating minimal bias between manual and automatic strain measurements. The standard deviation of the differences was 1.33%, suggesting a good level of agreement. The average left atrial strain across methods was 18.2%, falling within a clinically expected range. These findings confirm that the proposed manual method yields measurements that are closely aligned with the automatic software.

In the overall cohort (*n* = 60), manual and automatic left atrial reservoir strain measurements demonstrated excellent agreement, with a Pearson correlation coefficient of 0.995, *p* < 0.0001, and an intraclass correlation coefficient of 0.988. The coefficient of variation was 7.3%, indicating low relative dispersion between methods and low variability. The standard error of measurement was 0.94%, and the minimal detectable change at 95% confidence was 2.61%, corresponding to 14.3% of the average strain value, indicating high precision and sensitivity to small clinical changes. Table 5. These findings confirm that the proposed manual method is highly consistent with automated analysis.

To evaluate whether the accuracy of the manual method for calculation of left atrial reservoir strain varied by clinical characteristics, subgroup analyses were conducted. Patients were stratified by left atrial size, ejection fraction, age, and sex, Table 6. The manual method showed excellent agreement with the automated measurements across all subgroups, Table 6. The best agreement (highest intraclass correlation coefficient and lowest error) was in patients with enlarged left atrium and those with preserved ejection fraction. The lowest intraclass correlation coefficient was in the ejection fraction <50% group (0.894), but still within the “good” reliability range. Only minor differences between sexes and age groups were observed—all subgroups showed excellent correlation and reliability.

To assess the reliability of the manual left atrial strain measurements, both inter- and intra-observer reproducibility were evaluated in a subset of 15 randomly selected cases. The intraclass correlation coefficient (ICC) was calculated using a two-way random-effects model with absolute agreement. Inter-observer agreement (M.L. and V.T.) demonstrated excellent reliability, with an ICC of 0.982, while intra-observer agreement (M.L.) was similarly high, with an ICC of 0.986. These results confirm that the proposed manual method for left atrial strain assessment is highly reproducible and consistent across different observers and repeated measurements.

Post hoc power analysis confirmed that the study was adequately powered. With 30 patients in each group and the observed correlation coefficients (r = 0.95 in the atrial fibrillation group and r = 0.94 in the sinus rhythm group), the calculated statistical power exceeded 99%, supporting the robustness of the agreement observed between manual and automated measurements.

## 4. Discussion

This study validates a novel manual method for calculating biplane left atrial reservoir strain, demonstrating strong agreement with automated speckle tracking analysis in patients with both atrial fibrillation and sinus rhythm. These findings have important implications for the echocardiographic assessment of left atrial function, particularly in settings where automated tools are unavailable or impractical—such as resource-limited environments, retrospective analyses, and studies requiring cross-platform compatibility.

### 4.1. Clinical Relevance of Left Atrial Reservoir Strain

Left atrial reservoir strain is increasingly recognized as a sensitive biomarker of left atrial function and an important tool for cardiovascular risk stratification. Unlike static parameters such as left atrial volume, strain offers a dynamic assessment of myocardial deformation and is less dependent on preload conditions [1,2,3]. Reduced left atrial strain has been strongly associated with elevated left ventricular filling pressures, subclinical diastolic dysfunction, and adverse clinical outcomes in patients with both heart failure with preserved ejection fraction (HFpEF) and reduced ejection fraction (HFrEF) [3,5,10,11].

In particular, left atrial strain has emerged as a valuable prognostic marker in heart failure with preserved ejection fraction (HFpEF), offering incremental value beyond conventional parameters such as the E/e′ ratio and left atrial volume index [5,12]. Recent studies have demonstrated that impaired left atrial strain predicts future heart failure hospitalizations, atrial fibrillation recurrence, and cardiovascular mortality in patients with preserved left ventricular function as well as in those with valvular heart disease [13,14,15,16].

In patients with atrial fibrillation, left atrial strain is often markedly reduced, reflecting underlying structural remodeling and fibrosis. It has been shown to predict rhythm outcomes following cardioversion and catheter ablation, and to correlate with the presence of left atrial appendage thrombus and stroke risk—independent of the CHA_2_DS_2_-VASc score [6,11,17,18,19].

### 4.2. The Need for Manual Methods

Despite the well-established clinical value of left atrial strain, its widespread use remains limited by the need for dedicated speckle tracking software, which is often vendor-specific, expensive, or incompatible with retrospective image datasets [7,9,20]. In addition, variability among vendors and inconsistencies in tracking algorithms can impair reproducibility across platforms, thereby limiting the generalizability of strain-based metrics in multicenter studies and collaborative research settings [21,22,23].

Our study addresses these limitations by validating a manual approach based on point-to-point linear measurements of atrial length during systole and diastole, allowing for the calculation of reservoir strain without the use of dedicated speckle tracking software. This method demonstrated excellent reliability, with Pearson correlation coefficients exceeding 0.94 in both rhythm groups and intraclass correlation coefficients (ICCs) greater than 0.92. These results align with previous studies supporting the feasibility of simplified manual or semi-automated strain analysis when performed using standardized imaging planes and protocols [12].

The biplane approach employed in this study enhances measurement accuracy by incorporating both apical four-chamber and two-chamber views, in alignment with the recommendations from the European Association of Cardiovascular Imaging and the American Society of Echocardiography [7,9].

### 4.3. Diagnostic Accuracy and Measurement Reproducibility

Our data demonstrate a low standard error of measurement (SEM), minimal detectable change (MDC), and acceptable coefficient of variation (CV), all indicative of high reproducibility and sensitivity to subtle functional changes. These performance metrics are comparable to those reported for commercially available software, suggesting that the manual method is not only feasible but also clinically reliable. Notably, the atrial fibrillation group exhibited narrower limits of agreement in the Bland–Altman analysis compared to the sinus rhythm group, which was consistent with the lower within-method repeatability coefficients observed in AF. This finding may be attributed to the lower overall strain values and reduced dynamic range in atrial fibrillation, which likely result in less variability and facilitate more consistent delineation of atrial deformation. In contrast, sinus rhythm is characterized by greater reservoir function and broader physiological variability, which may contribute to the slightly increased dispersion in strain measurements.

The high correlation across the entire cohort (r = 0.995) and an intraclass correlation coefficient (ICC) of 0.988 confirm the robustness of the manual method, irrespective of underlying rhythm or pathology. These values exceed widely accepted thresholds for reproducibility in cardiovascular imaging [24], supporting the integration of this approach into both clinical practice and research settings.

Subgroup analysis demonstrated that the manual strain method showed excellent agreement with automated measurements in patients with enlarged left atria (ICC = 0.996) and those with preserved ejection fraction (ICC = 0.992). These results may be attributed to improved endocardial visibility and clearer contour distinction in enlarged atria, which facilitate more consistent manual annotation. Additionally, patients with preserved systolic function typically exhibit more uniform and robust atrial deformation patterns, enabling more precise manual frame selection.

In contrast, patients with normal left atrial size exhibited slightly higher variability (ICC = 0.981; mean difference = 1.56%), likely due to subtler deformation and less distinct anatomical landmarks. Similarly, patients with reduced ejection fraction demonstrated slightly lower—but still good—agreement (ICC = 0.894), possibly reflecting greater variability in atrioventricular coupling and atrial mechanics. Despite these differences, the manual method maintained good-to-excellent reproducibility across all subgroups, highlighting its clinical applicability in a wide range of cardiac conditions.

### 4.4. Broader Implications for Practice and Research

In clinical echocardiography laboratories with limited access to dedicated strain analysis software, or in studies involving archived echocardiographic data, this manual method offers a practical alternative that can be implemented without the need for reprocessing or costly post hoc analysis tools. Because digital calipers are universally available on all ultrasound systems, the approach is broadly accessible and easy to integrate into routine workflows.

Moreover, as multicenter studies increasingly incorporate strain imaging for patient phenotyping, vendor-neutral techniques are essential for ensuring consistency across platforms. Manual strain measurement may be particularly valuable for harmonizing datasets in institutions using different imaging systems. Prior studies have reported inter-vendor variability in left atrial strain values of up to 5–10%, which may significantly impact interpretation if not addressed [21,22].

While manual measurement is inherently more time-consuming, it offers greater transparency and operator control, allowing experienced clinicians to directly assess and verify each measurement. This is especially advantageous in cases with poor image quality, foreshortened views, or abnormal atrial anatomy, where automated tracking may fail.

Finally, this method may be particularly useful in specific clinical populations where left atrial function serves as a key diagnostic and prognostic marker. These include patients with heart failure with preserved ejection fraction, atrial myopathy, valvular heart disease, and those at risk for cardioembolic stroke. Integrating left atrial strain with biomarkers, volumetric indices, and clinical variables may enhance composite risk stratification models for cardiovascular events [25,26,27,28].

### 4.5. Study Limitations

This study has several limitations. First, it was conducted at a single center with a relatively modest sample size, which may limit the generalizability of the findings. Second, manual strain analysis may be influenced by image quality and operator experience; therefore, the use of standardized acquisition protocols and adequately trained readers is essential to ensure reproducibility.

The study was designed as a methodological validation and agreement analysis. Previous echocardiographic validation studies have demonstrated that sample sizes of 25–30 subjects per group are sufficient to detect strong agreement using correlation and intraclass correlation coefficient (ICC) analysis [29]. Nonetheless, we acknowledge the need for future investigations involving larger and more diverse populations to confirm the external validity of our findings.

Prior studies have shown that left atrial reservoir strain is independently associated with left ventricular global longitudinal strain, left atrial volume, and left ventricular filling pressures [18]. Additionally, increased left atrial sphericity has been linked to reduced reservoir strain and strain rate, particularly in patients with atrial fibrillation [30]. These structural and functional variables were not evaluated in the present study and fall beyond its scope.

Despite these limitations, our findings provide a strong proof-of-concept and offer a practical, accessible methodology for left atrial strain assessment using basic echocardiographic tools, supporting broader clinical and research applications.

## 5. Conclusions

In summary, our findings support the validity and reproducibility of a simple manual method for left atrial reservoir strain assessment. This technique demonstrates excellent agreement with automated software and offers a reliable alternative in situations where proprietary tools are unavailable. Broader adoption of such manual methods may enhance the utility of left atrial strain across diverse clinical and research environments, promoting equitable access to this valuable marker of atrial function.

## Figures and Tables

**Table 1:** Demographic and clinical characteristics of patients with atrial fibrillation, Group 1, and patients with normal sinus rhythm, Group 2.

	Group 1	Group 2	*p*-Value
Number	30	30	1.0
Males	19 (63.3%)	19 (63.3%)	1.0
Females	11 (36.7%)	11 (36.7%)	1.0
Age, years	79.1 ± 8.7	47.4 ± 20.9	<10^−9^
Height, cm	167.2 ± 8.3	170 ± 8.6	0.21
Weight, kg	79.2 ± 15.4	75.5 ± 13.4	0.21
BMI, kg/m^2^	28.3 ± 5.0	25.7 ± 3.7	<0.03
BSA, m^2^	1.88 ± 0.2	1.85 ± 0.2	0.57
NYHA FC	2.5 ± 0.8	1.1 ± 0.3	<10^−10^
Hypertension	22 (73.3%)	5 (16.7%)	<10^−5^
Diabetes	11 (36.7%)	3 (10%)	0.01
IHD	12 (40%)	0	0.0001
CKD	12 (40%)	1 (3.3%)	<0.01
COPD	12 (40%)	2 (6.7%)	<0.01
Malignancy	3 (10%)	7 (23.3%)	0.17

BMI—body mass index, BSA—body surface area, NYHA FC—New York Heart Association Functional Class of heart failure, IHD—ischemic heart disease, CKD—chronic kidney disease, COPD—chronic obstructive pulmonary disease.

**Table 2:** Echocardiography characteristics of patients with atrial fibrillation, Group 1, and patients with normal sinus rhythm, Group 2.

	Group 1	Group 2	*p*-Value
LVEDD, cm	4.6 ± 0.6	4.6 ± 0.5	0.83
LVESD, cm	3.1 ± 0.8	2.7 ± 0.4	0.02
EF, %	47.8 ± 10.5	60.5 ± 1.5	<10^−7^
IVST, cm	1.3 ± 0.4	1.0 ± 0.2	<10^−4^
PWT, cm	1.1 ± 0.2	0.9 ± 0.1	<10^−3^
LVMi, g/m^2^	109.3 ± 35.7	78.7 ± 21.2	<10^−3^
RWT	0.48 ± 0.14	0.40 ± 0.10	<0.01
LAD, cm	4.5 ± 0.7	3.6 ± 0.4	<10^−7^
LAA, cm^2^	30.1 ± 5.6	18.1 ± 3.8	<10^−12^
LAVi, mL/m^2^	56.7 ± 19.7	31.1 ± 10.4	<10^−7^
LASr, %	6.4 ± 2.3	30.3 ± 4.68	<10^−31^
LASr(m), %	6.5 ± 2.2	29.5 ± 4.4	<10^−32^
PAP, mmHg	44.2 ± 11.0	25.2 ± 6.5	<10^−10^
MR ≥ 2 degree	16 (53.3%)	0	<10^−4^
AS ≥ 2 degree	4 (13.3%)	0	<0.04
AI ≥ 2 degree	1 (3.3%)	0	0.32
TR ≥ 2 degree	20 (66.7%)	1	<10^−7^

LVEDD—left ventricle end diastolic diameter, LVESD—left ventricle end systolic diameter, EF—ejection fraction, IVST—interventricular septum thickness, PWT—posterior wall thickness, LVMi—left ventricular mass index, RWT—relative wall thickness, LAD—left atrial diameter, LAA—left atrial area, LAVi—left atrial volume index, LASr—left atrial reservoir strain measured automatically, LASr(m)—left atrial reservoir strain measured manually, PAP—pulmonary artery pressure, MR—mitral regurgitation, AS—aortic stenosis, AI—aortic regurgitation, TR—tricuspid regurgitation.

**Table 3:** Pearson correlation between manual and automatic methods.

Patients	LASr(m)	LASr	r	*p*-Value
AF	6.53 ± 2.2	6.4 ± 2.3	r = 0.953	*p* < 0.0001
Sinus	29.5 ± 4.35	30.3 ± 4.68	r = 0.939	*p* < 0.0001

AF—atrial fibrillation, LASr(m)—left atrial reservoir strain measured manually, LASr—left atrial reservoir strain measured automatically.

**Table 4:** Summary of correlation and agreement between manual and automatic methods for left atrial strain calculation.

Metric	Atrial Fibrillation Group	Sinus Group
Mean Manual Strain, %	6.5 ± 2.2	29.5 ± 4.4
Mean Automatic Strain, %	6.4 ± 2.3	30.3 ± 4.7
Mean Difference	0.09 ± 0.71	−0.80 ± 1.64
Upper Limit of Agreement	1.49	2.41
Lower of Agreement	−1.30	−4.01
Pearson Correlation (r, *p*-value)	0.953, *p* < 0.0001	0.939, *p* < 0.0001
Interclass Correlation	0.95	0.92
Coefficient of Variation for Manual-Automatic Differences	11.0%	5.5%
Standard Error of Measurement, %	0.16	0.46
Minimal Detectable Change (95), %	0.43	1.27
Repeatability Coefficient for manual method, %	0.82 (0.60–1.29)	10.53 (7.71–16.61)
Repeatability Coefficient for automatic method, %	0.7 (0.51–1.10)	5.66 (4.15–8.93)

**Table 5:** Agreement and reliability data for manual vs. automatic left atrial strain measurements across the study population (*n* = 60).

Pearson Correlation (r)	0.995
*p*-value	<10^−60^
Intraclass Correlation Coefficient	0.988
Coefficient of Variation (CV%)	7.32%
Standard Error of Measurement (SEM)	0.94%
Minimal Detectable Change (MDC95)	2.61%
MDC as % of mean	14.34%

Very high correlation and ICC indicate excellent agreement between manual and automatic methods. Low SEM and MDC indicate high precision and sensitivity to small clinical changes. CV of ~7.3% is acceptable and shows low relative variability.

**Table 6:** Subgroup analysis of manual versus automated left atrial reservoir strain calculation. LAVi—left atrium volume index, ICC—intraclass correlation.

Subgroup	N	Mean Absolute Difference, %	Pearson r	ICC
Left atrium enlarged (LAVi > 34 mL/m^2^)	36	0.73	0.996	0.996
Normal left atrium (LAVi ≤ 34 mL/m^2^)	24	1.56	0.986	0.981
Ejection fraction preserved	48	1.16	0.994	0.992
Ejection fraction reduced	12	0.65	0.907	0.894
Age ≥ 65	36	0.78	0.997	0.993
Age < 65	24	1.28	0.989	0.985
Male	38	1.02	0.996	0.994
Female	22	1.12	0.994	0.994

## Data Availability

The data supporting the findings of this study are available from the corresponding author upon reasonable request.
